# Comparative Analysis of RNAi-Based Methods to Down-Regulate Expression of Two Genes Expressed at Different Levels in *Myzus persicae*

**DOI:** 10.3390/v8110316

**Published:** 2016-11-19

**Authors:** Michaël Mulot, Sylvaine Boissinot, Baptiste Monsion, Maryam Rastegar, Gabriel Clavijo, David Halter, Nicole Bochet, Monique Erdinger, Véronique Brault

**Affiliations:** 1 Université de Strasbourg, INRA, SVQV UMR-A 1131, 28 rue de Herrlisheim, Colmar, 68021 Strasbourg, France; michael.mulot@colmar.inra.fr (M.M.); sylvaine.boissinot@colmar.inra.fr (S.B.); baptiste.monsion@supagro.inra.fr (B.M.); rastgarmaryam@gmail.com (M.R.); gaclavijo@hotmail.com (G.C.); david.halter@inra.fr (D.H.); nicole.bochet@colmar.inra.fr (N.B.); monique.erdinger@colmar.inra.fr (M.E.); 2 INRA, UMR BGPI INRA-CIRAD-SupAgro, CIRAD TA-A54/K, Campus International de Baillarguet, 34398 Montpellier, France; 3 Plant Protection Department, Shiraz University, Shiraz, Iran

**Keywords:** gene silencing, functional validation, aphids

## Abstract

With the increasing availability of aphid genomic data, it is necessary to develop robust functional validation methods to evaluate the role of specific aphid genes. This work represents the first study in which five different techniques, all based on RNA interference and on oral acquisition of double-stranded RNA (dsRNA), were developed to silence two genes, *ALY* and *Eph*, potentially involved in polerovirus transmission by aphids. Efficient silencing of only *Eph* transcripts, which are less abundant than those of *ALY*, could be achieved by feeding aphids on transgenic *Arabidopsis thaliana* expressing an RNA hairpin targeting *Eph*, on *Nicotiana benthamiana* infected with a *Tobacco rattle virus* (TRV)-Eph recombinant virus, or on in vitro-synthesized *Eph*-targeting dsRNA. These experiments showed that the silencing efficiency may differ greatly between genes and that aphid gut cells seem to be preferentially affected by the silencing mechanism after oral acquisition of dsRNA. In addition, the use of plants infected with recombinant TRV proved to be a promising technique to silence aphid genes as it does not require plant transformation. This work highlights the need to pursue development of innovative strategies to reproducibly achieve reduction of expression of aphid genes.

## 1. Introduction

Aphids are small sap-sucking insects belonging to the *Hemiptera* order. They display important demographic potential and adapt easily to changes in environmental conditions, causing significant damage to crops by direct feeding. Furthermore, they are vectors of numerous deleterious plant viruses, and are considered as one of the main animal pests for agriculture [1,2,3]. Several methods, mainly pesticide use, have been deployed in the field to reduce aphid populations, but the development of insecticide resistance has been an increasing problem for agriculture [3,4,5]. Additionally, the French Ecophyto 2018 plan [6], a program launched at the “Grenelle of Environment” which is aimed at reducing the use of inputs by 50% by 2018, could not be reached in the proposed period and was recently extended to 2025. In order to develop novel non-chemical methods to decrease aphid populations, or to reduce virus transmission, new molecular targets need to be discovered. The genomes of several aphid species, *Acyrthosiphon pisum* [7], *Myzus persicae* [8], and *Diuraphis noxia* [9] are now available, which offers a tremendous opportunity to identify genes with vital functions, or which are involved in virus transmission. Bioinformatic annotation assigns a putative function to a gene, but functional validation is required for confirmation. Functional validation is usually performed by inhibiting the expression of a gene and linking a specific phenotype to a gene function. Alternatively, over-expression of a candidate gene can be applied, particularly for genes belonging to multigenic families for which inhibition of one gene can be masked by the redundant function of the other family members. RNA interference (RNAi)-based strategies have been developed in aphids to conduct gene functional validation [10,11,12]. RNAi is based on the detection of double-stranded RNA (dsRNA) molecules by the cell machinery, which are diced into small interfering RNAs (siRNA) of 21–25 nucleotides. Each siRNA serves as a guide strand for the degradation of all RNA molecules sharing sequence-specific homology to the siRNA [13,14,15,16,17]. dsRNA or mature siRNA can activate RNA degradation in insect cells, and different methods to deliver these molecules into whole aphids have been employed [12,18]. Aphids can be fed on dsRNA or siRNA sources, such as (1) in vitro-synthesized dsRNA [19,20,21,22,23,24,25,26]; (2) siRNA synthesized chemically or obtained after enzymatic cleavage of the dsRNA [27,28]; or (3) plants stably or transiently expressing dsRNA [29,30,31,32,33,34]. An alternative method to deliver dsRNA or siRNA into aphids is by microinjection, although this technique is not well adapted to a large scale analysis of genes [19,22,27,28,30,35,36,37]. Regardless of the delivery method (oral acquisition or microinjection), genes expressed in the gut, at the salivary gland level, in the embryos, the head, or the carcass can be affected [10]. The non-cell autonomous effect of RNAi was confirmed by tracking distribution of fluorescently-labeled dsRNA in the aphid’s body after oral acquisition [24,26]. Importantly, persistent down-regulation of target genes was observed in the progeny of aphids that have been fed on transgenic plants expressing dsRNA [38].

Although several RNAi methods have been successfully applied, either to investigate aphid physiology, or to manage aphid populations, no description of any method have been so far reported to show the function of candidate genes in virus transmission by aphids. Moreover, the increasing literature on RNAi in aphids exemplifies the high degree of variability in silencing efficiency and expression inhibition seems to vary depending on the targeted gene and the method used to deliver dsRNA into the aphids. More surprisingly, silencing efficiency of a similar gene using the same method was also shown to vary depending on the laboratories conducting the experiments [19].

In this study, we sought to compare different RNAi-based silencing techniques in *M. persicae* all based on oral acquisition of long dsRNA, RNA hairpin, or siRNA. The two genes, *ALY* and *Eph*, potentially involved in polerovirus (family *Luteoviridae*) transmission by aphids were selected. Indeed, both encoded proteins displayed the ability to bind to the structural proteins of two aphid-transmitted poleroviruses in yeast (unpublished data) [39]. To confirm the role of these two genes in polerovirus transmission by aphids, it is necessary to efficiently and reproducibly inhibit expression of these genes in aphids before addressing the ability of these aphids to transmit the virus. ALY is a mRNA export factor which shuttles between the nucleus and the cytoplasm [40] and Eph plays a role in the maintenance of epithelial tissue homeostasis [41]. Both are expressed throughout the aphid body and in the gut, but *ALY* transcripts accumulated at a higher level compared to *Eph* transcripts. We were able to silence *Eph* after feeding aphids (1) on transgenic *Arabidopsis thaliana* expressing an RNA hairpin; (2) on *Nicotiana benthamiana* infected with a *Tobacco rattle virus* (TRV)-recombinant virus; or (3) on artificial medium containing in vitro synthesized dsRNA. In contrast, we were unable to reproducibly achieve silencing of *ALY*.

## 2. Materials and Methods

### 2.1. Aphid Rearing

Colonies of *M. persicae*, and *M. persicae* ssp. *nicotianae* were reared, respectively, on pepper (*Capsicum annuum*) or tobacco (*Nicotiana tabacum*) at 20 °C with a 16 h photoperiod.

### 2.2. Plant Material

*N. benthamiana* were grown in greenhouses for four weeks before being agroinfiltrated. Agroinfiltrated plants were grown in an environment-controlled chamber at 23 °C during the day and 20 °C during the night with a 10 h photoperiod. *A. thaliana* were grown in growth chambers with the same aforementioned setup.

### 2.3. Constitutive and Transient Expression of RNA Hairpins in Plants

Selection of the targeted sequences used in this study was made using the E-RNAi webtool (3.2 version) [42]. The transcript sequences from *A. pisum* (based on ACYPI accessions) were used to select the target region for *M. persicae*. LacZ fragment was determined using “sequence input” method with the full-length gene from pUC18. A fragment of 249 bp (nt 1198 to 1446 on the *Eph* coding sequence, homologous to the *A. pisum* gene accession number ACYPI064034-RA) was amplified by reverse transcription (RT) polymerase chain reaction (PCR) on total *M. persicae* RNA extracted using a commercial purification kit (RNeasy Plant Mini Kit, animal tissue protocol, QIAGEN, Hilden, Germany). The reverse primer contained BamHI and SwaI restriction sites, and the forward primer XbaI and AscI. The amplified DNA fragment was introduced in the sense and antisense orientations into the pFGC5941 vector (GenBank accession AY310901.1) in a sequential process: the PCR-amplified DNA fragment was first digested with AscI and SwaI and introduced into the pFGC5941 digested with the same enzymes. The recombinant plasmid bearing the sense sequence was further digested with XbaI and BamHI, and the PCR-amplified and enzyme-digested *Eph*-cDNA antisense sequence was introduced into this plasmid leading to pFGC:Eph. A similar procedure was followed to introduce a 182 bp fragment of the coding sequence of *ALY* (homologous to the *A. pisum* gene accession number ACYPI006176-RA) in sense and antisense orientations into the pFGC5941 vector. The *ALY* cDNA fragment was amplified by reverse transcription polymerase chain reaction (RT-PCR) from *M. persicae.* As a control, a 276 bp fragment from the *lacZ* gene was amplified by PCR from the pUC18 vector (GenBank accession L09136.1). The resulting plasmids were respectively referred to as pFGC:ALY and pFGC:LacZ. A complete list of primers used throughout the experiment is provided in Supplementary Materials Table S1. The pFGC:Eph, pFGC:ALY and pFGC:LacZ plasmids were further introduced into *Agrobacterium tumefaciens* C58C1.

To constitutively express the RNA hairpin sequences, pFGC:Eph, pFGC:ALY, and pFGC:LacZ were used to transform *A. thaliana* (Col-0) by floral dip as described by Martinez-Trujillo et al. [43]. T0 seeds were sown, and seedlings sprayed with phosphinothricin at 300 mg/L (BASTA^®^ F1; Bayer, Leverkusen, Germany) to select positive transformants. Subsequent generations (T1 to T3) were produced by selfing and selection with BASTA^®^ F1. Insertion of the transgene in the BASTA^®^ F1 resistant plants was verified by PCR on fresh tissue using the KAPA3G Plant PCR Kit (Kapa Biosystems, Wilmington, MA, USA), but the number of insertions was not determined. In the experiments using the T1 progeny of transgenic plants expressing an RNA hairpin targeting *ALY* or *Eph*, *A. thaliana* transformed with an RNA hairpin targeting the *green fluorescent protein* (*GFP*) gene were used as control [32].

To transiently express the RNA hairpin sequences in *N. benthamiana*, *A. tumefaciens* harboring the pFGC-derived plasmids were grown to an optical density (OD) of 0.5 at 600 nm and agroinfiltrated into fully-expanded leaves of 4–6 week old plants [44].

### 2.4. *Tobacco Rattle Virus* (TRV)-Derived Constructs and Inoculation

TRV has a bipartite genome split between RNA1 and RNA2. TRV-derived constructs referred to as pTRV1 and pTRV2 were used to induce the synthesis of dsRNA and siRNA in *N. benthamiana*. PCR-amplified DNA fragments corresponding to partial sequences of *Eph* (249 bp), *ALY* (182 bp) or *lacZ* (276 bp) were obtained as described above using primers listed in Table S1. The resulting PCR products were cloned in the sense orientation into BamHI/XbaI-cut pTRV2 [45] resulting in pTRV2-Eph, pTRV2-ALY, and pTRV2-LacZ. pTRV1 and pTRV2 derivatives were introduced into *A. tumefaciens* C58C1. The *Agrobacterium* cultures containing the TRV1-construct or the TRV2-derived plasmids were grown to an OD of 0.5 at 600 nm and mixed in equal proportions before infiltration of 4–6 week old *N. benthamiana*. Plant infection with TRV was confirmed by DAS-ELISA [46] using TRV-specific antibodies (Sediag, Bretenière , France).

### 2.5. Detection of Small Interfering RNA (siRNA) by Northern Blot and Small RNAs Purification for Aphid Acquisition

To evaluate siRNA accumulation in transgenic *A. thaliana* or in the agroinoculated leaves of *N. benthamiana*, total RNA was extracted from leaves using TRIzol™ Reagent (Sigma, St. Louis, MO, USA). Ten micrograms of total RNA were mixed with an equal volume of deionized formamide and denatured for 5 min at 95 °C. Total RNAs were resolved on a 17.5% polyacrylamide gel (19:1 acrylamide-bisacrylamide ratio, 7 M urea in Tris-Borate-EDTA buffer 0.5X) and blotted onto an Amersham Hybond-NX membrane (GE Healthcare, Chicago, IL, USA) by liquid transfer (Bio Rad, Criterion Blotter, Hercules, CA, USA) for 75 min at 80 V/300 mA. RNAs were fixed on the membrane by UV crosslinking (120,000 µJ/cm^2^, Spectrolinker XL-1000 UV Crosslinker; Spectronics, Westbury, NY, USA). DNA probes were prepared by PCR amplifications of 249 bp fragment for *Eph*, 182 bp fragment for *ALY* and 276 bp fragment for *lacZ* using primers listed in Supplementary Materials Table S1. In a second step, the PCR products were further labeled using a Klenow fragment (Roche, Basel, Switzerland) with digoxigenin (DIG) dUTP (Roche). Alternately, *ALY*-probe was labeled with ^32^P. To ensure equal loading of RNA, blots were hybridized with a probe targeting the U6 RNA (Supplementary Materials Table S1). The signals were detected using the chemiluminescence kit (CDP-Star; Roche) when using a DIG-labeled probe, or by autoradiography when using the radioactive probe.

The small RNAs were further precipitated from total RNA before delivery to aphids by membrane feeding. The RNA pellet from the Trizol extraction procedure was washed three times with 70% ethanol to eliminate toxic phenol traces before being suspended in RNase-free water. An equal volume of 20% PEG 8000 was then added to the total RNA suspension together with 1/10 volume of NaCl 5M. After a one hour incubation on ice, the mixture was centrifuged at 13,000 rpm for 10 min at 4 °C. The supernatant containing the small RNAs was mixed with three volumes of 100% ethanol before incubation at −20 °C for 2 h. After centrifugation at 13,000 rpm for 30 min at 4 °C, the pellet was washed 3 times with ethanol 70%, then resuspended in RNase-free water. Integrity of the small RNAs was checked by diluting in an equal volume of formamide and running at 50 V in TAE 0.5X.

### 2.6. In Vitro-Synthesized double-stranded RNA (dsRNA)

In vitro dsRNA fragments were produced using the RiboMAX™ Large Scale RNA Production System-T7 Kit (Promega, Madison, WI, USA) on PCR fragments made from LITMUS 28i recombinant vectors (New England Biolabs, Ipswich, MA, USA). The recombinant plasmids were obtained after introducing a 249 bp *Eph* fragment, a 182 bp *ALY* fragment, or a 276 bp *lacZ* fragment, synthesized by RT-PCR of total RNA extracted from *M. persicae* for the Eph and ALY constructs, or by PCR of pUC18 for the LacZ construct. These PCR fragments were digested by BamHI and XbaI before being introduced into LITMUS 28i digested with the same enzymes. To synthesize dsRNA, the recombinant LITMUS 28i vectors were used as templates to synthesize PCR fragments containing the introduced sequences flanked by T7 promoters at both extremities using T7 primers. PCR fragments were then purified with MSB™ Spin PCRapace system (Stratec, Birkenfeld, Germany) and used as template for in vitro transcription. The transcription mixtures containing both sense and anti-sense RNAs were denatured at 96 °C for 5 min and annealing of both RNA strands was performed by a progressive decrease to room temperature. RNAse H and DNAse Q1 treatments were subsequently performed on dsRNA for 20 min at 37 °C to remove DNA templates and single-stranded RNA. dsRNA were further purified using the MEGAclear™ Transcription Clean-Up Kit, (Thermo Fisher Scientific Ambion^TM^, Austin, TX, USA), spectrophotometrically quantified at 260 nm (Nanodrop 2000; Thermo Fisher Scientific), subjected to agarose gel electrophoresis to determine purity and stored at −20 °C before use.

### 2.7. dsRNA and/or siRNA Acquisition by *M. persicae*

For all the delivery methods, the acquisition time was selected in order to reach the maximum uptake of dsRNA and/or siRNA while maintaining a good survival rate of the aphids. These acquisition times varied, therefore, between the different dsRNA and/or siRNA sources.

On transgenic *A. thaliana* expressing hairpin constructs, fourth instar or adult individuals of *M. persicae* were deposited on the transgenic plants for two days. Adults were then removed, and nymphs born on the transgenic plants were kept for 10 to 13 additional days before collection for RNA extraction and real-time reverse transcription polymerase chain reaction (qRT-PCR) analysis.

From *N. benthamiana* infiltrated with the hairpin constructs, acquisition of siRNA or RNA hairpin by *M. persicae* ssp. *nicotianae* was performed by placing 1^st^ instar for 10 days on leaf discs cut from infiltrated leaves. The leaf discs were placed on top of 1% water agarose in small Petri dishes covered with a mesh, and were replaced with fresh infiltrated (five or six days post-infiltration) leaf discs every three days during a 10-day period to ensure consistent high levels of siRNA throughout the experiment.

Acquisition of small RNAs extracted from the aforementioned infiltrated leaves of *N. benthamiana* was done by feeding 4th instars or adults of *M. persicae* onto an artificial medium containing the small RNA fraction diluted in sucrose (20% final concentration) for a period ranging from 24 h to three days. Younger aphid stages did not survived onto this preparation.

*M. persicae* ssp. *nicotianae* (2nd instars) were deposited on *N. benthamiana* detached leaves from TRV-infected plants that were first washed and gently rubbed on their surface [47]. This leaf treatment improved aphid survival until seven days after deposition, when aphids started to decline and were collected for analysis. For *M. persicae* acquisition of dsRNA synthesized in vitro, 4th instars or adults were artificially fed for 72 h on dsRNA diluted in sucrose (20% final concentration) or in MP148 [47] placed between two Parafilm M™ layers. For each experimental condition, approximately 150 to 200 individuals were placed in a single black feeding chamber.

All experimental devices i.e. plant material infested with aphids, or aphids fed artificially, were placed in a containment growth chamber at 23 °C day and 20 °C night with a 10 h photoperiod.

### 2.8. RNA Extraction from Whole Aphids or Guts

Total RNA was extracted from whole *M. persicae* (8–20 aphids were pooled in each sample) using the RNeasy Plant Mini Kit (QIAGEN) following the procedure for animal tissue. Aphids were first ground with a pestle in the RLT lysis buffer in Eppendorf tubes (Eppendorf, Hamburg, Germany). RNA was eluted in 50 μL of RNase-free water. Total RNA was also extracted from dissected guts. Dissection was performed by first immobilizing aphids on double-sided tape and then pulling with tweezers the aphid’s head which comes along the foregut, the anterior midgut, and part of the posterior midgut. After removing the heads, 100 guts were pooled and collected in cold RLT lysis buffer from RNeasy Plant Mini Kit (QIAGEN). Total RNA was extracted as described above, except that grinding with pestles was avoided and the tube was placed in dry ice before RNA purification. RNA was eluted in 24 μL of RNase-free water and quantified at 260 nm with the Nanodrop 2000 (Thermo Fisher Scientific). These batches of RNA corresponding to several aphids or several guts extracts were reverse transcribed and analyzed by real-time PCR.

### 2.9. Real-Time Reverse Transcription Polymerase Chain Reaction (qRT-PCR) and Biological Repeats

For qRT-PCR, complementary DNA (cDNA) was first synthesized using oligo (dT) 15 Primer (Promega) with the M-MLV reverse transcriptase kit (Promega) starting from 200–1000 ng of total RNA from guts or whole aphids, respectively. qRT-PCR reactions were performed in triplicates in 96-well optical plates using 10 to 50 ng of cDNA, 0.6 µL of each primer at 10 mM, and 10 µL of SYBR™ Green Supermix (Bio-Rad, Hercules, CA, USA), in a final volume of 20 µL. The primers used to amplify *ALY*- or *Eph*-mRNA were located outside the target sequences to avoid amplification in the aphids of the acquired dsRNA. The qRT-PCR reactions were conducted on a CFX cycler (Bio-Rad) initiated with a 3 min incubation at 90 °C, followed by 40 cycles of amplification (10 s at 95 °C, 30 s at 60 °C). Melt curve analysis was performed from 60 °C to 95 °C with 5 s of 0.5 °C increments. Threshold cycle (CT) values were calculated using Bio-Rad CFX Manager^TM^ software (Bio-Rad). Relative expression levels were normalized to *Rpl7* [35] and *L27* [28] (Supplementary Materials Table S1) by subtracting the control CT values from CT values of *Eph*/*ALY*, to yield the ΔCT. The stability of *Rpl7* and *L27* expression was verified in five batches of 15 *M. persicae* adults or three batches of 80–90 digestive tubes collected from adults (in whole aphids, mean Cq values for *rpl7*: 20.27 ± 0.08 or *L27*: 16.28 ± 0.07 and in guts, mean Cq values for *rpl7*: 20.54 ± 0.07 or *L27*: 16.65 ± 0.05). The specificity of PCR primers was assessed by melting curve analysis of PCR products, and average amplification efficiency was determined by Bio-Rad CFX Manager^TM^ software. The relative expression levels of *Eph* and *ALY* were calculated using the ΔΔCT method. The results were analyzed for significant differences with Student’s *t*-test in which *n* = 3 refers to technical triplicates.

For all different RNAi methods, biological repeats were conducted when a reduction of the expression of the target gene was observed at least once. When the method resulted in an overexpression of the target gene or in instability of the reference genes for the four conditions tested (*Eph* and *ALY*, whole aphids, and guts), it was not systemically repeated.

## 3. Results

### 3.1. Selection of the Candidate Genes to be Silenced in *M. persicae*

**Figure 1:**
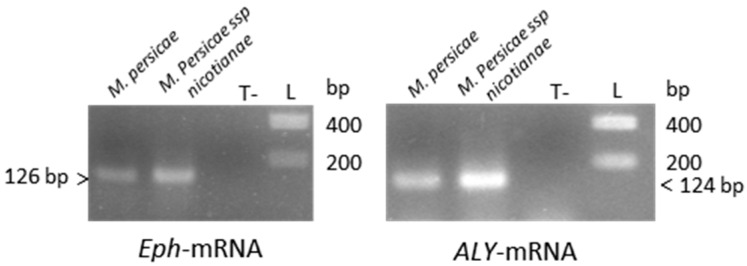
Detection of *ALY*- and *Eph*-mRNA in *M. persicae* (Sulzer) or in *M. persicae* ssp. *nicotianae* by reverse transcription polymerase chain reaction (RT-PCR; Supplementary Materials Table S1). PCR amplified fragments were loaded on a 1.5% agarose gel and visualized by UV after ethidium bromide staining. This detection is not quantitative since different amounts of RNA have been used in the RT-PCR reactions. T-: negative control, L: DNA ladder.

In order to evaluate the efficacy of different RNAi-based silencing techniques in aphids, we selected two genes from *M. persicae*, *Eph* and *ALY*, that are potentially involved in polerovirus transmission. To confirm the function of these genes in the aphid transmission process, functional validation is required which can be addressed by analyzing virus transmissibility of aphids in which expression of these genes is inhibited. *ALY* transcripts accumulated at a higher level compared to *Eph* mRNA in non-viruliferous *M. persicae*, which provided the opportunity to compare the efficacy of the silencing techniques with two genes that have different baseline levels of transcript abundance. *Eph,* a homolog of the Mammalian Ephrin receptor (Eph), encodes a membranous tyrosine kinase receptor and is involved in cell communication, clathrin-mediated endocytosis, and cellular trafficking [48]. *ALY* encodes a nuclear protein known to be involved in mRNA trafficking between nucleus and cytoplasm [40,49]. RNA sequencing data analysis from Aphidbase revealed that *ALY*-mRNA was about eight-fold and 75-fold more abundant in whole aphids and gut cells, respectively, compared to *Eph*-mRNA. These data were obtained from female adults of a different aphid clone than the one used in the present study. We however verified the higher accumulation of *ALY*-mRNA compared to *Eph*-mRNA in whole aphids and in guts by qRT-PCR using the *M. persicae* clone reared in the laboratory (data not shown). Expression of *ALY* and *Eph* was also confirmed by RT-PCR in *M. persicae* ssp. *nicotianae*, another clone of *M. persicae* used in some of the following experiments (Figure 1).

The aphid gene sequences that were targeted by RNAi met several criteria as the specificity for the target gene in *M. persicae* and a length of about 200 bp. *ALY* has a short coding sequence (801 bp) and the target sequence of 182 bp corresponds to 22.7% of this sequence. On the other hand, *Eph* is a larger gene (about 3200 bp) which mRNA is subjected to alternative splicing, giving rise to different variants [50]. The target sequence of 249 bp is included in a central sequence shared by all *Eph*-mRNA and represents around 7.7% of the coding sequence. None of the selected sequences share a perfect homology of more than 14 bp with the genomic sequences of *A. thaliana* or *N. benthamiana*. These sequence homologies are not sufficient to induce silencing of genes in these two plant species. A 276 bp sequence from the *lacZ* gene served as a control in all RNAi-based experiments. This bacterial sequence does not share any identity with the genome of either *M. persicae* or the aphid primary symbiont *Buchnera aphidicola*. The target sequences have different GC content, 35.0%, 51.2%, and 55.0% for *ALY*, *Eph* and *lacZ*, respectively, which could account for differential stability of the dsRNA.

### 3.2. Acquisition of dsRNA or siRNA from Plants Constitutively Expressing RNA Hairpins

**Figure 2:**
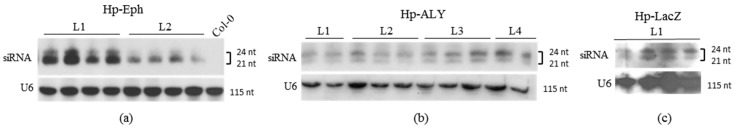
Small interfering RNA (siRNA) detection in *A. thaliana* (**a**) Hp-Eph; (**b**) Hp-ALY and (**c**) Hp-LacZ. Total RNA was extracted from four-week old seedlings of the T1 progeny of transgenic *A. thaliana*. Each lane was loaded with 20 µg of total RNA and the blots were hybridized with digoxigenin (DIG)-labeled probes, except in (**b**), where radioactive probes were used to detect U6 RNA and *ALY*-siRNA. The other blots were further stripped and hybridized with a DIG-labeled U6 probe as a loading control. Col-0: total RNA extract from a non-transformed *A. thaliana* Col-0 plant.

One of the strategies employed to inhibit the expression of *ALY* and *Eph* genes in aphids was the feeding of aphids on plants constitutively expressing RNA hairpin structures targeting the genes of interest. This strategy has already been validated in numerous studies with aphids [29,31,32,33,34,38,51,52,53]. The fragment of 249 bp from the *Eph* coding sequence was introduced in sense and antisense orientations into the pFGC5941 vector. These two sequences were separated by the chalcone synthase intron sequence, and the whole construct was placed under the control of the 35S promoter of *Cauliflower mosaic virus* (CaMV). A similar strategy was followed to introduce the 182 bp inverted tandem sequence of *ALY* into pFGC5941. These recombinant plasmids were used to transform *A. thaliana* by floral dip, and the resulting transgenic plants were referred to as Hp-ALY and Hp-Eph. Transcription of the introduced sense and antisense sequences in the transgenic plants is expected to result in the synthesis of RNA hairpin structures that can further be processed into siRNA by the silencing machinery of the plants. Control plants designed to produce the 276 bp RNA-hairpin sequence targeting the *lacZ* gene (plants named Hp-LacZ) were also engineered. Processing of the RNA hairpin into siRNA was further analyzed by northern blot in two to four plants from independent lines of the T1 (Figure 2) or T3 (not shown) progenies. Presence of small RNAs derived from the hairpin sequences was observed in lines transformed with Hp-Eph, Hp-ALY and Hp-LacZ, although variability in siRNA accumulation was sometimes observed between lines. In particular, Hp-Eph plants from line 1 of the T1 progeny accumulated a slightly higher level of siRNA compared to line 2 (Figure 2a). Specificity of each probe was controlled on RNA extracts from non-transformed Col-0 plants (Figure 2a for Eph-probe; not shown for other probes). Overall, we observed that all of the transgenic plants designed to express RNA hairpins accumulated siRNA corresponding to the introduced hairpins with no major differences between the different progenies (not shown).

To address whether aphid feeding on the transgenic plants Hp-Eph or Hp-ALY expressing RNA hairpins could induce a reduction of *Eph*-mRNA or *ALY*-mRNA accumulation, *Eph*- and *ALY*-mRNA accumulation was measured by qRT-PCR on *M. persicae* fed on these plants. Although inhibition of *Eph* expression was reproducibly observed in aphids fed on plants from the T1 progeny, no such consistent results were obtained when using the T3 progeny (Table 1A). It is interesting to point out that the silencing efficiency of *Eph* was more pronounced in aphids fed on line 1 plants of Hp-Eph, which is also the line that accumulated more siRNA (Figure 2a). Surprisingly, some experiments showed an over-accumulation of *Eph*-mRNA in aphids fed on Hp-Eph (for example, 12% over-expression in one replicate of Exp. 6, Table 1A). These unexpected data may correspond to a genuine increase of *Eph*-mRNA, as it was already reported 24 h after acquisition of dsRNA targeting the *Cathepsin-L* gene in *A. pisum* [22], but could also represent intrinsic variability between biological samples. Taking into account this potential variability in the results, we thereafter considered a true inhibition of gene expression when the mRNA accumulation of the targeted gene was at least reduced by 25%.

When aphids feed on transgenic *A. thaliana*, the orally acquired long dsRNA or processed siRNA must be first internalized into *M. persicae* intestinal cells. We, therefore, addressed whether the inhibition of *Eph* expression could also be observed in aphid guts. *Eph*-mRNA accumulation was measured specifically in the gut cells after dissecting the aphid guts seven days after feeding on Hp-Eph. This experiment was performed on aphids fed on the T3 or T4 progenies of Hp-Eph. Although no consistent silencing was observed in whole aphids fed on T3 transgenic lines, an 87% decrease of *Eph*-mRNA accumulation was observed at the gut level (Table 2A). It should be mentioned that the same batch of aphids was used to analyze gene expression in whole insects and in guts. This result suggests that inhibition of *Eph* expression is probably effective in aphids fed on the T3 progeny of Hp-Eph but at a level which can only be observed when analyzing aphid guts. However, no such sustained inhibition of *Eph* expression at the gut level was obtained when feeding aphids on the T4 progeny (Table 2A), suggesting a variability in the silencing efficiency between plant generations.

When aphids were fed on Hp-ALY plants, no visible down-regulation of *ALY* was observed in whole aphids or in guts (Table 3A and Table 4A).

### 3.3. Acquisition of dsRNA/siRNA from Plants Expressing Transiently RNA Hairpins

**Figure 3:**
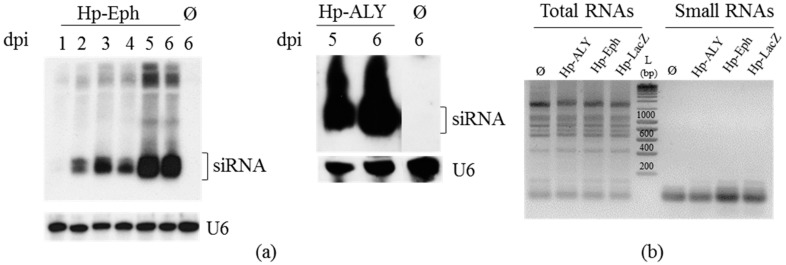
(**a**) siRNA detection and purification from *N. benthamiana* transiently expressing Hp-Eph and Hp-ALY. siRNA were extracted at different days after infiltration (dpi) of *N. benthamiana* leaves with *Agrobacterium* carrying pFGC5941-derived plasmids expressing hairpin RNAs targeting *Eph* or *ALY*. Each lane was loaded with 10 µg of PEG-purified small RNAs and the blots were hybridized with DIG-labeled probes. The blot on the left was further stripped and hybridized with a DIG-labeled U6 probe as loading control, whereas the U6-probe was added together with the ALY-probe in the blot on the right. The two panels of this blot were manually organized (probe detection placed under siRNA) for consistency. Ø: Non-infiltrated *N. benthamiana* leaves; (**b**) 2.5% agarose gel loaded with 1.0 to 1.8 µg of total RNAs extracted from *N. benthamiana* leaves five days after infiltration with *Agrobacterium* bearing pFGC5941-derived plasmids expressing hairpin RNAs targeting *Eph*, *ALY* and *lacZ*. Small RNAs were purified with PEG and used for aphid feeding; 0.8 to 1.0 µg of small RNAs were loaded on the gel. The gel was stained with ethidium bromide. The size (in bp) of the DNA ladder (L) is indicated.

The second strategy we used to decrease *Eph* and *ALY* expression was the transient expression in *N. benthamiana* of RNA hairpins targeting these two aphid genes. The recombinant plasmids pFGC:Eph and pFGC:ALY previously used to stably transform *A. thaliana* were introduced into *N. benthamiana* leaves by agroinfiltration. As shown in Figure 3a, a high accumulation of siRNA was observed 5–6 days after infiltration. Aphid acquisition of siRNA or RNA hairpin was performed using *M. persicae* ssp. *nicotianae* clone since these aphids are well adapted to *N. benthamiana*, unlike *M. persicae* for which we observed a high mortality on this plant species. Unfortunately, the real-time RT-PCR data obtained from whole aphids fed for 10 days on *N. benthamiana* transiently expressing Hp-Eph or Hp-ALY could not be used due to unstable values of the reference genes between different samples (Table 1B and Table 3B). Unexpectedly, such variation in the reference gene values was not observed in guts dissected from these aphids, but no down-regulation of either *Eph*- or *ALY*-mRNA was observed (Table 2B and Table 4B).

In order to increase the amount of siRNA potentially acquired by aphids from the infiltrated leaves, small RNAs were purified from leaves infiltrated with one of the following constructs, pFGC:Eph, -ALY or -LacZ, five days after agroinfiltration. As shown in Figure 3b, enriched fractions of small RNAs were recovered from total RNAs, which contained a mixture of small RNAs, including siRNA and miRNAs. These small RNA-enriched samples were delivered to aphids by membrane feeding. Aphid viability was affected when feeding on this medium and we, therefore, tested different concentrations of small RNAs in the aphid diet (from 60 to 120 ng/µL) and different acquisition times (24 h to 3 days) to select the optimal conditions for this assay. Using a concentration of small RNAs of 100 ng/µL and a 72 h-acquisition time (conditions kept for the other experiments), we obtained a good aphid survival rate, but did not obtain consistent silencing of *Eph* in whole insects (Table 1C). Important modifications in the aphid metabolism probably occurred when feeding for three days on purified small RNA preparations targeting *Eph* or *ALY*, since no exploitable data of the reference genes were obtained for any of the conditions tested (Table 1D, Table 2D, Table 3D and Table 4D). From these experiments, we can conclude that delivering significant amounts of small RNAs to aphids may induce perturbations of aphid metabolism that are not compatible with accurate measurement of transcripts accumulation.

### 3.4. RNAi-Based Silencing by Feeding Aphids on Plants Infected with a TRV-Recombinant Virus

**Figure 4:**
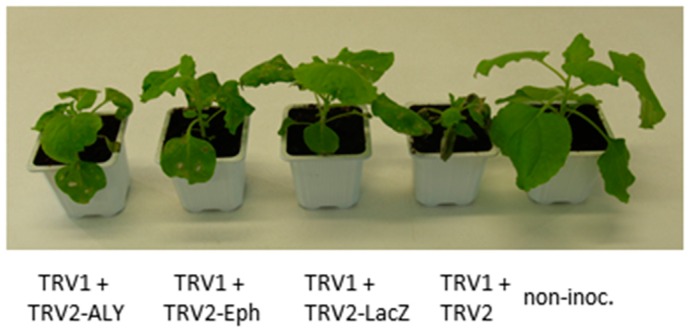
Development of *N. benthamiana* infected with recombinant *Tobacco rattle virus* (TRV). *N. benthamiana* eight days after infiltration with a mixture of *Agrobacterium* containing TRV1 and TRV2 constructs, or one of the following TRV2-derivatives: TRV2-Eph), TRV2-ALY, or TRV2-LacZ. non-inoc.: non-inoculated *N. benthamiana*.

Double-stranded RNA viral replication intermediates can initiate the RNAi mechanism, ultimately resulting in the production of siRNA targeting the whole viral genome. Therefore, introducing a foreign sequence into the viral genome can lead to the production of siRNA targeting the introduced sequence and also any mRNA sharing homology with this sequence, a mechanism referred to as virus-induced gene silencing (VIGS) [54,55]. Partial sequences from *Eph*, *ALY* or *lacZ* genes were inserted into TRV-RNA2, and different recombinant TRV-RNA2 constructs were agroinfiltrated in *N. benthamiana* together with TRV1 [45]. Plant infection with TRV was confirmed by DAS-ELISA four weeks post-inoculation and the presence of the inserted sequence in the viral progeny was also verified by RT-PCR on total RNA extracted from non-inoculated leaves using specific primers (data not shown). *N. benthamiana* plants inoculated with TRV1 and one of the following recombinant constructs TRV2-Eph, TRV2-ALY, or TRV2-LacZ displayed milder symptoms compared to plants inoculated with TRV1 and the non-recombinant TRV2 (Figure 4). This observation has already been reported in tomato inoculated with TRV-derived constructs and seems to be linked to the introduction of exogenous sequences in TRV2 [56]. As in previous studies, however, no change in virus accumulation measured by DAS-ELISA was observed (data not shown).

Interestingly, when *M. persicae* ssp. *nicotianae* (second instar nymphs) were deposited on recombinant TRV-infected *N. benthamiana*, *Eph*-mRNA accumulation was reduced in whole aphids by 41.9% in two independent experiments (Table 1C) and by 18.8% and 55.9% in the aphid guts (Table 2C). The level of *ALY* silencing in aphids fed on *N. benthamiana* infected with TRV-ALY was much lower in whole aphids (11.9% and 15.3% reduction of *ALY*-mRNA) compared to the silencing obtained for *Eph* and was not reproducibly obtained in aphid guts (Table 3C and Table 4C).

### 3.5. RNAi-Based Silencing by Artificial Feeding on In Vitro Synthesized dsRNA

**Figure 5:**
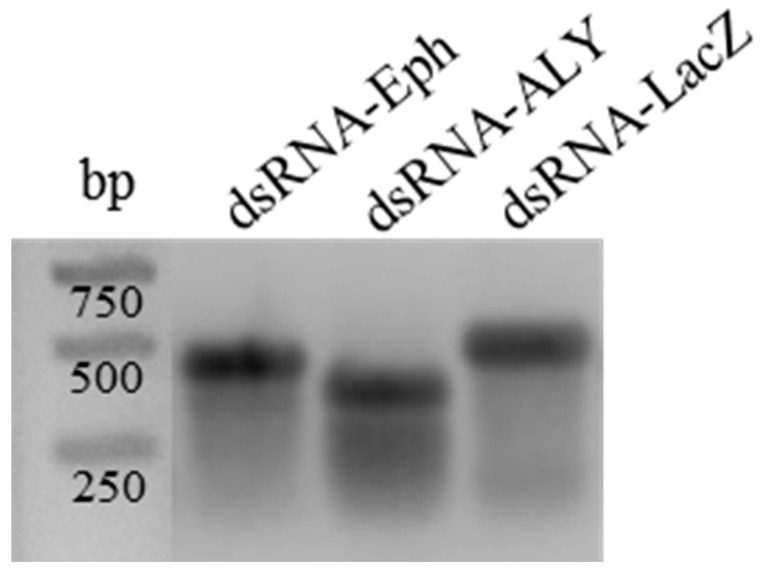
Electrophoretic mobility of in vitro synthesized double-stranded RNA (dsRNA). 800 ng of dsRNA-Eph, -ALY, and -LacZ synthesized in vitro were deposited on a 1% agarose gel stained with ethidium bromide. Sizes (in bp) of the molecular markers are indicated. Due to the double-stranded structure of the RNA, their mobility is reduced.

In vitro-synthesized dsRNA of 249 bp for *Eph*, 182 bp for *ALY*, and 276 bp for *lacZ* (Figure 5), were delivered to aphids in an artificial medium. When using a 72 h acquisition period and a concentration of 100 ng/µL for the dsRNA, no significant reduction of *Eph*-mRNA accumulation (8.3% to 9.8% inhibition) was obtained in whole aphids (Table 1E). By raising the dsRNA concentration in the aphid diet to 200 and 400 ng/µL, a 53% and 84.7% of *Eph*-mRNA inhibition was observed in the aphid guts, respectively (Table 2E). Silencing of *ALY* was not observed in whole aphids fed for three days on an artificial medium containing 400 ng/µL of dsRNA targeting *ALY* (Table 3E), but when we analyzed the aphid guts of aphids from the same experimental set-up, a high silencing of *ALY* was obtained in one experiment (73% of *ALY* expression inhibition) out of the two performed (Table 4E). The discrepancy between these two experiments may be attributed to the gut dissection procedure that could release cellular contents and potentially inhibitory components affecting the qRT-PCR reaction.

## 4. Discussion

RNAi-based techniques have previously been developed to conduct functional validation in several aphid species. However, the reduction of accumulation of the targeted gene transcripts varies considerably depending on the gene and the method used to deliver dsRNA to aphids. This significant variability in RNAi efficiencies complicate the choice of a functional validation technique when analyzing the function of a selected gene. Our main objective was to compare the efficiency of several RNAi methods for their capacity to inhibit the expression of two genes in *M. persicae*, *ALY* and *Eph*, which are potentially involved in polerovirus transmission by aphids, but for which a functional validation assay is required to ascertain their function. Even though candidate genes have been identified as potential virus receptors in aphids [57,58,59,60], no functional validation was yet reported, likely due to the difficulty to obtain a significant and reproducible inhibition of the candidate genes in aphids. In particular, the identification of the first receptor for *Pea enation mosaic virus* (family *Luteoviridae*) in *A. pisum*, the alanyl aminopeptidase N, was based on in vitro experiments and in vivo competition experiments between the virus and a peptide potentially mimicking the viral determinant binding the aphid receptor [61,62]. The discovery of candidate genes reinforces the necessity to assess the efficacy of functional validation methods to confirm the role of virus receptor candidates in aphids. The work described in this paper is, however, not a comprehensive exploration of all technical means to achieve gene silencing in aphids, as several methods potentially able to target an aphid gene were not included in the analysis (microinjection of synthetic siRNA or dsRNA and oral acquisition of synthetic siRNA, for example). However, this is the first study in which several silencing methods were assessed in a single laboratory using the same genes, similar aphid clones, and assessment criteria (qRT-PCR), thus eliminating a number of potentially significant sources of variation. In addition, all methods targeted the same region of each gene which corresponds to part of the coding sequence. Additionally, oral acquisition of the silencing molecules (long dsRNA, RNA hairpin, or siRNA) offers the possibility to adapt the silencing strategy to large scale analyses of aphid genes.

### 4.1. Oral Acquisition of dsRNA/siRNA from Transgenic Plants, from Plants Infected with Recombinant TRV, and from In Vitro-Synthesized Transcripts Are the Most Efficient Gene Silencing Methods in Aphids

Among the five different RNAi techniques assessed, the most efficient methods to silence *Eph* were the feeding of aphids (1) on transgenic *A. thaliana* expressing an RNA hairpin targeting *Eph*; (2) on *N. benthamiana* infected with TRV-Eph; and (3) on in vitro-synthesized transcripts derived from *Eph* sequences. However, we observed differences in the silencing efficiency induced by the three techniques. Reproducible silencing was obtained in whole aphids when they were fed on Hp-Eph plants from the T1 progeny. In contrast, no consistent silencing of *Eph* was obtained when using the following progeny (T3). Surprisingly, this difference was not correlated with a low accumulation of siRNA in the T3 plants suggesting that the long dsRNA, rather than the siRNA, may be the molecules priming silencing. A size requirement of more than 60 bp for the dsRNA molecules to achieve efficient silencing of the target gene was already observed in western corn rootworm [63]. Silencing of *Eph* was particularly efficient at the gut level when the aphids were fed on T3 plants, but not when they were placed on T4 plants, suggesting that the silencing efficiency may vary greatly between plant generations. When feeding aphids on in vitro synthesized dsRNA targeting *Eph*, an efficient silencing was reproducibly observed at the gut level and not in whole aphids. These data suggest that the gut cells, and more likely the non-chitinous midgut and hindgut cells, are the preferential sites for dsRNA uptake and for efficient RNA silencing. These observations also point out that the silencing signal may not be well amplified and/or not efficiently transferred to neighboring cells. Although genes coding for the complete silencing machinery have been identified in the genome of the aphid *A. pisum* [64], phylogenetic analyses found that aphids lack the RNA-dependent RNA polymerase (RdRp), an enzyme responsible for signal amplification during RNAi [65,66]. The mechanism of RNAi signal spread is unknown in insects but there is strong evidence that this mechanism occurs in aphids [38]. Interestingly, *Eph* silencing was reproducibly obtained in whole aphids and in the guts after feeding the insects on *N. benthamiana* infected with TRV-Eph. To our knowledge, this technique has never been tested before to silence genes in aphids, but was successfully developed to silence genes in some phloem feeding insects as the potato psyllid *Bactericera cockerelli*, and the mealybugs *Planococcus citri* and *Phenacoccus solenopsis* [67,68,69]. In these previous experiments, a *Tobacco mosaic virus-* or a *Potato virus X*-derived virus was used to silence the insect genes. The use of a viral vector to generate silencing molecules that can be taken over by aphids is a very promising technique, as it does not require plant transformation and is potentially not affected by the silencing variations observed between plant progenies. Moreover, it is less expensive than in vitro dsRNA synthesis.

Silencing of *ALY* was more challenging. Reduction of *ALY*-mRNA accumulation was only found in the gut cells and only after feeding aphids on in vitro synthesized transcripts targeting *ALY*. Again, using *N. benthamiana* infected with a TRV-recombinant virus targeting *ALY* was the only way to observe a moderate silencing in whole aphids.

Taken together, these experiments show that the efficiency of silencing may depend greatly on the level of expression of a target gene. However, several other factors can also influence the silencing efficiency as the size of the dsRNA, the primary sequence and the secondary structure of the mRNA that can affect accessibility by the RISC complex, the temporal and tissue specificity expression of the gene, and the turnover of transcripts. The stability of the dsRNA in the aphid digestive tubes can also be a criteria for the success of silencing and, in this respect, the lower GC content of ALY-dsRNA could result in a higher degradation in the gut lumen compared to Eph-dsRNA. In addition, plant dicer-like proteins (DCLs) seem to operate preferentially on GC-rich region which would be in favor of a higher silencing activity on *Eph* [70]. The percentage of coverage of the target gene by the dsRNA does not seem to impact the gene silencing efficiency since the dsRNA targeting *ALY*-mRNA covers a larger portion of the coding sequence compared to *Eph*, without inducing a higher gene inhibition. Another observation deduced from our data is that the gut cells, which are the first to be targeted by dsRNA after oral acquisition, are more responsive to silencing than the other parts of the aphid body.

### 4.2. Feeding Aphids on *N. benthamiana* Transiently Expressing Hairpin RNAs, or on Small RNAs from These Plants, Are Not Efficient Silencing Methods

The other two techniques tested, which consisted of feeding aphids on *N. benthamiana* transiently expressing hairpin RNAs, or on siRNA purified from these plants, resulted in a particularly inefficient, or non-reproducible, silencing of *ALY* or *Eph*. Several reasons can be put forward to explain this result. Firstly, in infiltrated leaves transiently expressing hairpin constructs, dsRNA or siRNA mainly accumulate in epidermal cells which are only briefly sampled during the short aphid probes. Ingestion of these cells for only a brief period of time may not be sufficient to induce a strong gene silencing in aphids. It should however be mentioned that Pitino et al. [32] obtained 30% to 40% inhibition of the accumulation of the gut gene *Rack-1* and the salivary gland gene *MpC002* gene after feeding aphids on agroinfiltrated leaves of *N. benthamiana*. Whether or not this difference is due to the expression level of the different genes targeted, or to other factors, needs to be addressed. Secondly, feeding aphids on small RNAs purified from agroinfiltrated leaves to concentrate the amount of siRNA acquired by the aphids may result in instability of the two reference genes used in the qRT-PCR analyses. It is likely that ingestion of high amounts of small RNAs may saturate the silencing machinery resulting in aphid metabolism perturbations. Probably for similar reasons, such aberrant values of the reference genes were sometimes observed after feeding aphids on *N. benthamiana* transiently expressing hairpin RNAs.

### 4.3. Future Developments

To increase the efficiency of dsRNA uptake by aphids, several strategies can be followed: First of all, expression of the dsRNA specifically in phloem cells, under the control of phloem-specific promoters, could potentially increase dsRNA or siRNA release in sieve tubes and thus their uptake by phloem-feeding insects [12]. This promising development is moreover strengthened by the detection of miRNA and siRNA in sieve tubes [71,72], suggesting an efficient loading of small RNAs from companion cells or phloem parenchyma cells into sieve tubes. Another alternative is the use of transfection reagents or coating reagents for dsRNA or siRNA to increase stability and internalization of these molecules in insect cells [25,73,74]. This technology has already been successfully developed to boost gene silencing in mosquitoes [75].

Despite the development of several strategies to silence genes in aphids, there is still an urgent need to further elaborate technological innovations to increase the silencing efficiency and the persistence of silencing in aphids. Introducing stable genomic modifications in aphids has already been achieved in *A. pisum* using non-targeted ethyl methansulfonate (EMS) [76], and future developments will focus on application of the increasingly popular CRISPR (clustered regularly interspaced short palindromic repeats)/Cas9 gene-editing tool in aphids [77]. Another promising technology to conduct functional validation in aphids is the use of a gene vector derived from an aphid virus. In this respect, the *Myzus persicae densovirus* [78,79], which possesses a small single-stranded DNA genome, represents a good candidate following similar work with a mosquito densovirus [80]. This functional virus-based technology was used to inhibit gene expression, but also to over-express a specific gene, which would represent a major breakthrough in aphid post-genomic research.

## Figures and Tables

**Table 1:** Silencing efficiency of *Eph* in whole *M. persicae* using different RNA interference (RNAi)-based methods.

	Aphid Feeding Source	Exp.	Plant or RNA Concentration	AAP ^b^	Relative Fold Change *Eph*-mRNA in Whole *M. persicae* ^c^
A	Transgenic *A. thaliana* expressing Hp-Eph ^a^	1	Ara:Hp-Eph (T1) line 1	10 d	0.248 ± 0.037 (−75.2%) ^d,^*
			Ara:Hp-Eph (T1) line 2		**0.873 ± 0.103 (−12.7%) ^d^**
		2	Ara:Hp-Eph (T1) line 1	10 d	0.140 ± 0.065 (−86.0%) ^d,^*
			Ara:Hp-Eph (T1) line 2		0.533 ± 0.089 (−46.7%) ^d,^*
			Ara:Hp-Eph (T1) line 2		0.428 ± 0.145 (−57.2%) ^d,^*
		3	Ara:Hp-Eph (T3)	10 d	0.818 ± 0.032 (−18.2%) *
					1.032 ± 0.024 (+3.2%)
		4	Ara:Hp-Eph (T3)	10 d	1.048 ± 0.028 (+4.8%)
					**1.027 ± 0.060 (−2.8%)**
		5	Ara:Hp-Eph (T3)	10 d	0.843 ± 0.049 (−15.7%) *
					1.065 ± 0.055 (+6.5%)
					0.914 ± 0.43 (−8.6%) *
		6	Ara:Hp-Eph (T3)	13 d	**0.882 ± 0.078 (−11.8%)**
					1.120 ± 0.068 (+12.0%)
					0.726 ± 0.034 (−27.3%) *
B	*N. benthamiana* transiently expressing Hp-Eph	1	Bentha:Hp-Eph	10 d	*unstable reference genes* ^f^
C	*N. benthamiana* infected with TRV-Eph	1	Bentha:TRV-Eph	7 d	0.581 ± 0.018 (−41.9%) *^,e^
		2			0.581 ± 0.012 (−41.9%) *
D	siRNA purified from *N. benthamiana* transiently expressing Hp-Eph	1	siRNA 60 ng/µL	24 h	1.143 ± 0.054 (+14.3%)
		2	siRNA 120 ng/µL	24 h	**0.856 ± 0.042 (−14.4%) ^NA^**
					**0.832 ± 0.048 (−16.8%) ^NA^**
		3	siRNA 70 ng/µL	36 h	**0.936 ± 0.077 (−6.3%)**
		4	siRNA 100 ng/µL	60 h	1.056 ± 0.060 (+5.6%)
		5	siRNA 100 ng/µL	72 h	*unstable reference genes*
E	In vitro-synthesized dsRNA-Eph	1	dsRNA 100 ng/µL	72 h	0.902 ± 0.013 (−9.8%) *
		2			**0.917 ± 0.092 (−8.3%)**

^a^ In brackets: *A. thaliana* T1 or T3 progeny; ^b^ Acquisition Access Period; ^c^ Relative fold change of *Eph*-mRNA accumulation ± standard deviation of triplicates; In brackets, the level of expression compared to aphids fed on control conditions (*A. thaliana* constitutively expressing dsRNA targeting *lacZ*, *N. benthamiana* transiently expressing dsRNA targeting*lacZ*, *N. benthamiana* infected with a recombinant TRV-LacZ, in vitro synthesized dsRNA-LacZ). Each result corresponds to one pool of 20 aphids; In bold, the samples of aphids in which silencing of *Eph* was observed; In grey, samples in which *Eph*-mRNA was reduced by more than 25%; ^d^ *A. thaliana* constitutively expressing dsRNA targeting GFP used as control; ^e^ In this experiment, only the *Rpl7* reference gene has been used because of instability of the *L27* gene; ^f^ unstable reference genes: *Rpl7* and *L27* genes were not expressed at the same level in aphids being subjected to different treatments; * indicates significant differences between aphids fed on LacZ control and aphids that have acquired silencing molecules targeting *Eph* (Student’s *t*-test, p < 0,05); ^NA^ Analysis of variance not applicable; Exp., experiment; d, days; h, hours; TRV, *Tobacco rattle virus*; dsRNA, double-stranded RNA; siRNA, small interfering RNA.

**Table 2:** Silencing efficiency of *Eph* in *M. persicae* guts using different RNAi-based methods.

	Aphid Feeding Source	Exp.	Plant or RNA Concentration	AAP ^b^	Relative Fold Change *Eph*-mRNA in *M. persicae* Guts ^c^
A	Transgenic *A. thaliana* expressing Hp-Eph ^a^	1	Ara:Hp-Eph (T3) Ara:Hp-Eph (T4)	7 d	0.126 ± 0.021 (−87.4%) *
		2			**0.928 ± 0.062 (−7.8%)**
B	*N. benthamiana* transiently expressing Hp-Eph	1	Bentha:Hp-Eph	10 d	1.038 ± 0.037 (+3.8%)
C	*N. benthamiana* infected with TRV-Eph	1	Bentha:TRV-Eph	7 d	0.812 ± 0.013 (−18.8%) *
		2			0.441 ± 0.037 (−55.9%) *
D	siRNA purified from *N. benthamiana* transiently expressing Hp-Eph	1	siRNA 100 ng/µL	72 h	*unstable reference gene* ^d^
E	In vitro-synthesized dsRNA-Eph	1	dsRNA 200 ng/µL	72 h	0.470 ± 0.021 (−53.0%) *
		2	dsRNA 400 ng/µL		0.153 ± 0.015 (−84.7%) *

^a^ In brackets: *A. thaliana* T3 or T4 progeny; ^b^ Acquisition Access Period; ^c^ Relative fold change of *Eph*-mRNA accumulation ± standard deviation of triplicates. In brackets the level of expression compared to aphids fed on control conditions (*A. thaliana* constitutively expressing dsRNA targeting *lacZ*, *N. benthamiana* transiently expressing dsRNA targeting *lacZ*, *N. benthamiana* infected with a recombinant TRV-LacZ, in vitro synthesized dsRNA-LacZ). Each result corresponds to one pool of 100 aphid guts. In bold, the samples of aphids in which silencing of *Eph* was observed. In grey, samples in which *Eph*-mRNA was reduced by more than 25%; ^d^ unstable reference genes: *Rpl7* and *L27* genes were not expressed at the same level in aphids being subjected to different treatments; * indicates significant differences between aphids fed on LacZ control and aphids that have acquired silencing molecules targeting *Eph* (Student’s *t*-test, *p* < 0.05).

**Table 3:** Silencing efficiency of *ALY* in whole *M. persicae* using different RNAi-based methods.

	Aphid Feeding Source	Exp.	Plant or RNA Concentration	AAP ^b^	Relative Fold Change *ALY*-mRNA in Whole *M. persicae* ^c^
A	Transgenic *A. thaliana* expressing Hp-ALY ^a^	1	Ara:Hp-ALY (T1)	10 d	0.862 ± 0.105 (−13.8%) ^d^ *
		2			1.177 ± 0.023 (+17.7%) ^d^ *
		3			1.040 ± 0.026 (+4.0%) ^d^
		4			1.079 ± 0.161 (+7.9%) ^d^
B	*N. benthamiana* transiently expressing Hp-ALY	1	Bentha:Hp-ALY	10 d	*unstable reference genes* ^e^
D	*N. benthamiana* infected with TRV-ALY	1	Bentha:TRV-ALY	7 d	**0.881 ± 0.033 (−11.9%)**
		2			0.847 ± 0.033 (−15.3%) *
C	siRNA purified from *N. benthamiana* transiently expressing Hp-ALY	1	siRNA 100 ng/µL	72 h	*unstable reference genes*
E	In vitro synthesized dsRNA-ALY	1	dsRNA 400 ng/µL	72 h	1.041 ± 0.037(+4.0%)

^a^ In brackets: *A. thaliana* T1 progeny; ^b^ Acquisition Access Period; ^c^ Relative fold change of *ALY*-mRNA accumulation ± standard deviation of triplicates. In brackets the level of expression compared to aphids fed on control conditions (*A. thaliana* constitutively expressing dsRNA targeting *lacZ*, *N. benthamiana* transiently expressing dsRNA targeting *lacZ*, *N. benthamiana* infected with a recombinant TRV-LacZ, in vitro synthesized dsRNA-LacZ). Each result corresponds to one pool of 20 aphids. In bold, the samples of aphids in which silencing of *ALY* was observed; ^d^ *A. thaliana* constitutively expressing dsRNA targeting GFP used as control; ^e^ unstable reference genes: *Rpl7* and *L27* genes were not expressed at the same level in aphids being subjected to different treatments. * indicates significant differences between aphids fed on LacZ control and aphids that have acquired silencing molecules targeting *ALY* (Student’s *t*-test, *p* < 0.05).

**Table 4:** Silencing efficiency of *ALY* in *M. persicae* guts using different RNAi-based methods.

	Aphid Feeding Source	Exp.	Plant or RNA Concentration	AAP ^b^	Relative Fold Change *ALY*-mRNA in *M. persicae* Guts ^c^
A	Transgenic *A. thaliana* expressing Hp-ALY ^a^	1	Ara:Hp-ALY (T3)	13 d	1.315 ± 0.055 (+31.5%) *
		2		13 d	1.062 ± 0.007 (+6,2%) *
B	*N. benthamiana* transiently expressing Hp-ALY	1	Bentha:Hp-ALY	10 d	1.073 ± 0.051 (+7.3%)
C	*N. benthamiana* infected with TRV-ALY	1	Bentha:Hp-ALY	7 d	1.136 ± 0.036 (+13.6%) *
		2			0.915 ± 0.023 (−8.5%) *
D	siRNA purified from *N. benthamiana* transiently expressing Hp-ALY	1	siRNA 100 ng/µL	72 h	*unstable reference genes* ^d^
E	In vitro-synthesized dsRNA-ALY	1	dsRNA 400 ng/µL	72 h	0.263 ± 0.023 (−73.7%) *
		2			**0.942 ± 0.039 (−5.8%)**

^a^ In brackets: *A. thaliana* T3 progeny; ^b^ Acquisition Access Period; ^c^ Relative fold change of *ALY*-mRNA accumulation ± standard deviation. In brackets the level of expression compared to aphids fed on control conditions (*A. thaliana* constitutively expressing dsRNA targeting *lacZ*, *N. benthamiana* transiently expressing dsRNA targeting *lacZ*, *N. benthamiana* infected with a recombinant TRV-LacZ, *in vitro* synthesized dsRNA-LacZ). Each result corresponds to one pool of 100 aphid guts. In bold, the samples of aphids in which silencing of *ALY* was observed. In grey, samples in which *ALY*-mRNA was reduced by more than 25%; ^d^ unstable reference genes: *Rpl7* and *L27* genes were not expressed at the same level in aphids being subjected to different treatments; * indicates significant differences between aphids fed on LacZ control and aphids that have the silencing molecules targeting *ALY* (Student’s *t*-test, *p* < 0.05).

## Supplementary Materials

The following are available online at www.mdpi.com/1999-4915/8/11/316/s1. Table S1: List of primers.
