# Supplementary material for: Comparative Analysis of RNAi-Based Methods to Down-Regulate Expression of Two Genes Expressed at Different Levels in Myzus persicae
Source: Viruses. 2016 Nov 19;8(11):316. doi: 10.3390/v8110316 (PMC5127030; doi:10.3390/v8110316)
Supplement: Supplementary file 1 [file viruses-08-00316-s001.docx]

Supplementary Materials: Comparative Analysis of RNAi-Based Methods to Down-Regulate Expression of Two Genes Expressed at Different Levels in
*Myzus persicae*

Michaël Mulot, Sylvaine Boissinot, Baptiste Monsion, Maryam Rastegar, Gabriel Clavijo, David Halter, Nicole Bochet, Monique Erdinger and Véronique Brault

**Table S1.** List of primers.

| **Primer Sequence 5′ > 3′** | **Position (on CDS  Sequence When Relevant)** | **Sense** | **Restriction Sites (Underlined)** | **Experiments** |
| --- | --- | --- | --- | --- |
| TAGGATCCATTTAAATAGATAGCGTGATCTCGGACG | 1427–1446 | Reverse | BamHI, SwaI | Constitutive expression in A. thaliana of hp-EPHR Transient expression in N. benthamiana of hp:EPHR pLitMUS28i-derived vector  TRV-derived vector  Control of transgene integration |
| TTTCTAGAGGCGCGCCGGTCCATCCGTTGTGTTCAT | 1198–1217 | Forward | XbaI, AscI | Constitutive expression in A. thaliana of hp-EPHR Transient expression in N. benthamiana of hp:EPHR pLitMUS28i-derived vector  TRV-derived vector  Control of transgene integration |
| CCGGATCCATTTAAATGCAGTTTTTAATGGTCCAAA | 391–410 | Reverse | BamHI, SwaI | Constitutive expression in A. thaliana of hp-ALY Transient expression in N. benthamiana of hp:ALY pLitMUS28i-derived vector  TRV-derived vector  Control of transgene integration  ALY probe for siRNA detection/transgene detection in plants |
| TGTCTAGAGGCGCGCCTTCTAAGGGTGATGTGAATG | 228–247 | Forward | XbaI, AscI | Constitutive expression in A. thaliana of hp-ALY Transient expression in N. benthamiana of hp:ALY TRV-derived vector  pLitMUS28i-derived vector  ALY probe for siRNA detection/transgene detection in plants |
| TAGGATCCATTTAAATTAGAGTCGACCTGCAGGCAT | from pUC18 | Reverse | BamHI, SwaI | Constitutive expression in *A. thaliana* of hp-LacZ Transient expression in *N. benthamiana* of hp:LacZ pLitMUS28i-derived vector  TRV-derived vector  Control of transgene integration |
| GTTCTAGAGGCGCGCCCGGCATCAGAGCAGATTGTA | from pUC18 | Forward | XbaI, AscI | Constitutive expression in A. thaliana of hp-LacZ Transient expression in N. benthamiana of hp:LacZ pLitMUS28i-derived vector  TRV-derived vector  Control of transgene integration |
| AGATAGCGTGATCTCGGACG | 1427–1446 | Reverse |  | Eph probe for siRNA detection/ trangene detection in plants |
| GGTCCATCCGTTGTGTTCAT | 1198–1217 | Forward |  | Eph probe for siRNA detection/ trangene detection in plants |
| TAGAGTCGACCTGCAGGCAT | from pUC18 | Reverse |  | LacZ probe for siRNA detection/ trangene detection in plants |
| CGGCATCAGAGCAGATTGTA | from pUC18 | Forward |  | LacZ probe for siRNA detection/ trangene detection in plants |
| AAATTTGGACCATTTCTC | 394–411 | Reverse |  | U6 control probe |
| ATTGTCCCTTCGGGGAC | 308–324 | Forward |  | U6 control probe |
| CCATGATTACGCCAAGCTACG | T7 promoter | Forward |  | in vitro transcription/dsRNA synthesis |
| ACGTTGTAAAACGACGGCCAG | T7 promoter | Reverse |  | in vitro transcription/dsRNA synthesis |
| ATGTTGGTAAAGGCGTCCGAGA | 1414–1435 | Forward |  | Eph-mRNA qRT-PCR detection  Eph-mRNA detection in *M. persicae nicotianae* |
| ACTCGTCACCTCGGGGATAGAAC | 1518–1540 | Reverse |  | Eph-mRNA qRT-PCR detection  Eph-mRNA detection in *M. persicae nicotianae* |
| ACTGTTGCTAATCTTGCTACAC | 562–583 | Forward |  | ALY-mRNA qRT-PCR detection  ALY-mRNA detection in *M. persicae nicotianae* |
| TGTTGCCACGAGTATTACCG | 666–685 | Reverse |  | ALY-mRNA qRT-PCR detection  ALY-mRNA detection in *M. persicae nicotianae* |
| gCGCGCCGAGGCTTAT * | 154–168 | Forward |  | RPL7 reference gene qRT-PCR detection |
| CCgGatTTCTTTGCATTTCTTG * | 221–233 | Reverse |  | RPL7 reference gene qRT-PCR detection |
| CCGAAAAGCTGTCATAATGAAGAC | 1812–1835 | Forward |  | L27 reference gene qRT-PCR detection |
| GGTGAAACCTTGTCTACTGTTACATCTTG | 2014–2042 | Reverse |  | L27 reference gene qRT-PCR detection |

* In lower case: mismatches when hybridization on the *M. persicae* sequence.
